# Correlation between the concentrations of lactoferrin and neutrophil gelatinase-associated lipocalin in meconium

**DOI:** 10.1007/s10534-017-0073-3

**Published:** 2017-12-29

**Authors:** Barbara Lisowska-Myjak, Ewa Skarżyńska, Paulina Wilczyńska, Artur Jakimiuk

**Affiliations:** 1 Department of Biochemistry and Clinical Chemistry, Medical University of Warsaw, ul. Banacha 1, 02-097 Warsaw, Poland; 2 Clinical Department of Obstetrics, Female Diseases and Gynecological Oncology, Central Clinical Hospital of the Ministry of the Interior, Warsaw, Poland

**Keywords:** Lactoferrin, Neutrophil gelatinase-associated lipocalin, Meconium, Fetus

## Abstract

Neutrophil gelatinase-associated lipocalin (NGAL) and lactoferrin (Lf) are among the key components of the innate immune system due to their ability to bind iron with high affinity and thus control inflammation. The aim of this study was to test the use of NGAL and LF measurements in meconium for the assessment of the intrauterine homeostasis. NGAL and Lf concentrations were measured using ELISA kits in all serial meconium portions (n = 81) collected from 20 healthy neonates. Mean ± SD meconium concentration of Lf was 45.07 ± 78.53 µg/g and more than 1000-fold higher compared with that of NGAL at 1.93 ± 2.46 ng/g. The correlation between the two proteins (r = 0.83, p < 0.0001) was found only for portions with Lf concentrations > 25 μg/g. High variability of NGAL and Lf concentrations in meconium and their correlations prove their key role as biomarkers of the fetal condition in utero. NGAL and Lf measured in meconium are candidate biomarkers for fetal iron status.

## Introduction

Current methods of prenatal laboratory diagnosis do not offer any biological markers which would allow to confirm whether the intrauterine environment is homeostatic and thus help to detect any disturbances in homeostasis with potential short- and long- term postnatal health consequences.

Lactoferrin (Lf) and neutrophil gelatinase-associated lipocalin (NGAL) are among the key components of the innate immune system and because of their excellent iron-carrying properties provide valuable protection against all types of injury (Pacora et al. 2000; Ward et al. 2003; Schmidt et al. 2007; Tadesse et al. 2011; Pandita et al. 2015). Co-localized in specific (secondary) granules of human neutrophils, Lf and NGAL are released as acute phase proteins following neutrophil activation and degranulation (Borregaard et al. 2007; Parrow et al. 2013).

Increased Lf and NGAL concentrations observed in bacterial infection, inflammatory disease and other forms of cellular stress have been linked to high affinity for Fe3+ exhibited by the two proteins. By removing iron from the site of injury Lf and NGAL may limit iron-mediated toxicity, although the mechanisms each employs differ (Yang et al. 2002; Pandita et al. 2015).

NGAL, a 25-kDa lipocalin, interacts with specific receptors as a complex it forms with iron-binding siderophores derived from both bacteria and metabolic conversion in aseptic tissue. NGAL delivers iron to cells, regulates its intracellular storage, provides a reservoir for excess iron, may supply a regulated source of intracellular iron to stimulate repair and regeneration, and promotes apoptosis after transporting iron to the extracellular space (Tadesse et al. 2011; Bao et al. 2015; Nasioudis and Witkin 2015).

Lf, an 80-kDa member of the transferrin family of iron-binding glycoproteins, is thought to contribute to local iron accumulation in the intercellular space and body fluids at the site of inflammation. Lactoferrin binds iron with higher affinity than transferrin and unlike NGAL which binds iron in a complex with non-heme compounds, lactoferrin simply binds free iron (Pacora et al. 2000; Ward et al. 2003; Pandita et al. 2015; Sharma and Shastri 2015).

It is still unclear whether Lf and NGAL could be used for the assessment of homeostasis in the intrauterine environment at different stages of fetal development. Zhang et al. identified NGAL and NGAL receptors in human embryos and fetuses in different stages of development and found that the expression of NGAL and NGALR was time-specific and highly tissue-specific (Zhang et al. 2012).

Meconium is a matrix on which multiple physiological and non-physiological agents from the intrauterine environment like swallowed amniotic fluid, shed mucosal cells, cellular degradation products, intestinal secretions and ingested maternal or fetal blood from the digestive tract vessels are deposited and can be subsequently assessed. It is a viscid, odourless, greenish-black material excreted within about 48 h after birth by full-term neonates and the change in consistency and colour to yellowish-brown marks the end of passing meconium and start of excreting faeces. Meconium is not a homogenous material but a series of layers formed in the fetal intestine starting at 12 weeks of gestation (Ostrea 2001; Lisowska-Myjak and Pachecka 2007). The aim of this study was to measure NGAL and Lf concentrations and their relationship in serial portions of meconium passed by full-term neonates.

## Patients, materials and methods

### Patients

Twenty neonates, born in the Clinical Department of Obstetrics, Female Diseases and Gynecological Oncology, Central Clinical Hospital of the Ministry of the Interior, Warsaw, between May and June 2012, were included in the study.

### Neonate profile

- *Gender* 8 females and 12 males
- *Gestational* age [weeks] mean ± SD: 38.5 ± 1.4 (range 36–41)
- *Birth weight* [g] mean ± SD: 3272 ± 575.7 (range 2040–4280)
- *Apgar Score* 1/3/5/10, 10/10/10/10 (n = 16); 9/10/10/10 (n = 1), 9/9/10/10 (n = 1); 8/9/10/10 (n = 1); 9/9/9/9 (n = 1)

### Material

The study material consisted of all consecutive meconium portions (n = 81) collected serially from 20 neonates. The meconium was collected from the nappy with a disposable spatula and transferred into 50-ml graduated plastic containers. The date, time and weight of each collection were recorded. The collected material was frozen at − 20 °C for approximately 3–5 days and subsequently stored at − 80 °C for up to 3 months.

Prior to protein assay, meconium portions were thawed over 12 h at 4 °C. Analytical grade distilled water was added to the container with meconium to a final homogenate volume of 45 ml. The material was thoroughly shaken using a 358S Shaker at 350 cpm in a horizontal position for 30 min. The homogenate was subsequently poured into 2-ml deep-freeze test tubes and stored at − 80 °C.

### Assay of proteins

Measurements of meconiun protein concentrations were performed according to the manufacturer’s instructions in a MINIBOS immunochemistry analyser (DiaSorin), using the following kits:

- *for lactoferrin* The Human Lactoferrin ELISA kit, Assaypro LLC
- *for NGAL* The NGAL ELISA kit, BioPorto Diagnostics

Concentrations of lactoferrin expressed as ng/ml homogenate were calculated as µg/g meconium. Concentrations of the NGAL expressed as pg/ml homogenate were then calculated as ng/g meconium. The measurements were performed in duplicate and the mean value calculated was reported as a final result.

### Statistical analysis

Statistical analysis was performed using the STATISTICA software, version 12.0. Results are reported as mean, standard deviation, median and range.

One-way analysis of variance (ANOVA) was used to assess the differences between groups of neonates. Correlations between meconium protein concentrations and between their total intestinal accumulation were assessed with the Spearman rank correlation. Statistically significant differences were assumed when p < 0.05.

## Ethical approval

A written informed consent was obtained from the parents of all the newborns before they were included in the study. The Local Human Research Ethics Committee at the Central Clinical Hospital of the Ministry of the Interior approved the study and the procedures were in accordance with the ethical standards.

## Results

The mean concentrations of Lf and NGAL in all portions of meconium (n = 81) passed postnatally by 20 healthy neonates are shown in Table 1.

**Table 1:** Meconium concentrations of Lf and NGAL (n = 81 portions)

Parameter	Mean ± SD (range)	Median (95% CI)
Lactoferrin (µg/g)	45.07 ± 78.53 (1.69–511.43)	18.98 (27.68–62.46)
NGAL (ng/g)	1.93 ± 2.46 (0.24–12.82)	1.02 (1.39–2.48)

*Nonparametric Spearman’s rank correlation coefficient r = 0.50, p = 0.0001

Marked intra- and inter-individual variability of the meconium concentrations of the proteins was demonstrated: up to 303-fold for Lf and 54-fold for NGAL. The correlation between Lf and NGAL concentrations (r = 0.50) was statistically significant (p < 0.0001).

**Fig. 1:**
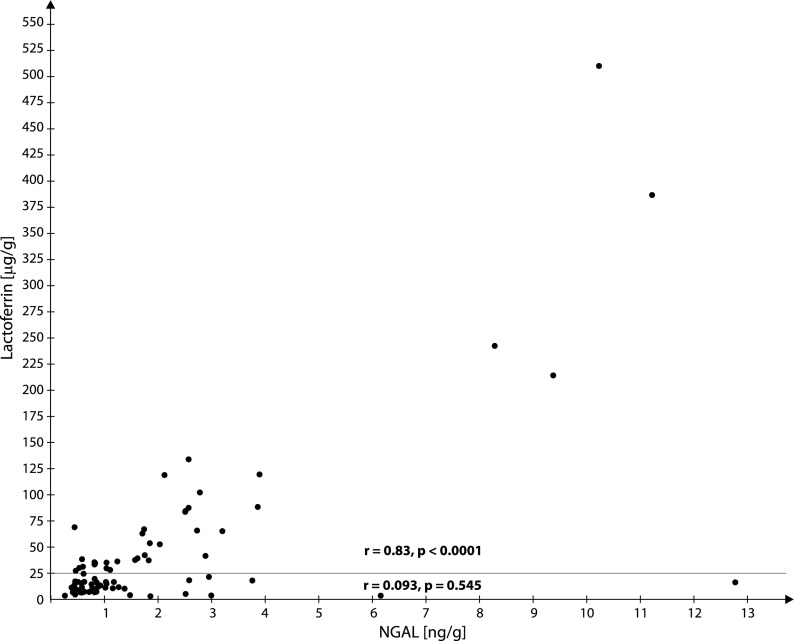
Graphical analysis of correlations between meconium concentrations of NGAL and lactoferrin. Differences between the correlation coefficients NGAL/lactoferrin for lactoferrin concentrations < 25 μg/g: r = 0.093, p < 0.545 for lactoferrin concentrations > 25 μg/g: r = 0.83, p < 0.0001

Figure 1 is the graphical analysis of the correlations between Lf and NGAL concentrations in 81 meconium portions. A statistically significant correlation between Lf and NGAL was demonstrated for 36 portions with Lf concentrations > 25 μg/g (r = 0.83, p < 0.0001) but in the remaining 45 portions with lower Lf concentrations (< 25 μg/g), the correlation was not statistically significant (r = 0.093, p = 0.545).

The total accumulation of Lf and NGAL in the intestine of the developing fetus was determined by the sum of these proteins in consecutive meconium portions. The term ‘meconium’ described a dark-greenish-black material with a characteristic gel-like consistency. Meconium collection was considered complete when on inspection the consistency and color had changed, the latter to the yellowish-brown characteristic of stool. Passing of meconium stopped between 14 and 52 h postnatally. One to nine meconium portions were collected from each neonate. The weight of a single meconium portion was [g]: mean ± SD = 4.52 ± 4.02 (range 0.19–18.93). The total weight of all meconium portions passed by an individual neonate was [g]: mean ± SD = 18.29 ± 8.64 (range 4.72–36.95).

The amounts of Lf [µg] and NGAL [ng] accumulated in the fetal intestine are presented in Table 2.

**Table 2:** Total accumulation of Lf and NGAL in the fetal intestinal contents (n = 20 neonates)

Parameter	Mean ± SD (range)	Median (95% CI)
Lactoferrin (µg)	757.3 ± 745.4 (20.5–2749.6)	514.7 (408.5–1106.1)
NGAL (ng)	27.73 ± 21.44 (1.88–71.70)	20.73 (1.88–71.70)

*Nonparametric Spearman’s rank correlation coefficient between lactoferrin and NGAL r = 0.65, p = 0.0018

A significant correlation was demonstrated between Lf and NGAL (r = 0.65, p = 0.0018).

Table 3 shows a statistically significant, nearly twofold increase in Lf and NGAL concentrations in consecutively passed meconium portions. For the purposes of the evaluation the weights of all consecutive portions of meconium passed by an individual neonate, i.e. 100% of the meconium accumulated in the fetus’s intestine during gestation, were summed up and then presented as the percentage of the total weight of meconium passed by all neonates.

**Table 3:** Lf and NGAL concentrations in consecutive meconium portions

Chronological sequence and % of the totasl meconium weight	n	Lactoferrin (µg/g) Mean ± SD median (range)*	NGAL (ng/g) Mean ± SD median (range)**	Correlation***
0–20	12	24.36 ± 21.86 18.97 (2.50–63.81)	1.41 ± 0.85 1.45 (0.36–3.00)	R = − 0.007, p = 0.98
20–40	14	31.94 ± 65.03 10.99 (1.69–248.87)	1.74 ± 2.40 0.90 (0.24–8.28)	R = − 0.024, p = 0.93
40–60	14	35.69 ± 37.51 25.21 (3.02–130.26)	1.10 ± 0.82 0.85 (0.39–2.59)	R = 0.691, p = 0.006
60–80	14	24.91 ± 21.26 15.90 (4.92–79.11)	1.14 ± 0.72 0.81 (0.48–2.52)	R = 0.407, p = 0.149
80–100	27	76.40 ± 118.96 25.13 (4.34–511.43)	3.11–3.54 1.83 (0.34–12.82)	R = 0.712, p < 0.0001

*Lactoferrin—differences between consecutive segments of the intestine (ANOVA, p = 0.154)

**NGAL—differences between consecutive segments of the intestine (ANOVA, p = 0.039)

***Nonparametrnic Spearman’s rank correlation coefficient (r)

In the first portions of meconium, i.e. up 40% of the total weight of meconium passed after birth, no correlation was found between Lf and NGAL concentrations. High correlations between the two were found in the later portions of meconium corresponding to 40–100% of its total weight.

## Discussion

Marked intra- and inter-individual variability of both NGAL and Lf concentrations in the meconium and their mutual correlations presented in this study suggests a decisive role of these proteins during the intrauterine development. Despite mean meconium concentration of Lf being over 1000-fold higher than that of NGAL a statistically significant correlation was found between two parameters. Additional graphical analysis, however, demonstrated that the correlation was found only for those meconium portions where the Lf concentrations exceeded 25 μg/g.

Based on the data from published studies, increases in meconium Lf and NGAL concentrations are most likely due to varied activation and degranulation of neutrophils in the intrauterine environment of the developing fetus. Variations in the levels of these proteins were analysed for each neonate in consecutive meconium portions assuming that the composition and level may change in response to changing intrauterine environment at particular stages of fetal development. Neutrophils are known to maintain homeostasis, utilizing their capacity for transformation from a dormant state to maximal metabolic and destructive activity at the site of inflammation (Borregaard et al. 2007).Gradual elevations in Lf and NGAL concentrations in consecutive meconium portions found in this study indicate that a fetus’s demand for Lf and NGAL and their functions increases with gestational age. A high correlation between Lf and NGAL concentrations in the last portions of meconium compared with the first portions leads to a logical conclusion that the increases are likely to be due to a common stimulus, the importance of which grows in the course of pregnancy. The finding of the presented neutrophil-derived proteins in the course of pregnancy suggested that it is possible to see additional markers of neutrophils, like myeloperoxidase to gain insights into the origin of NGAL.

Both Lf and NGAL are capable of binding ferric iron (Fe3+). NGAL, however, binds the Fe3+ -siderophore complex, while Lf chelates iron directly. The knowledge of the specific role Lf and NGAL have in the regulation of iron trafficking between the extra- and intracellular space may help to elucidate the differences in the relationship between meconium concentrations of the two proteins demonstrated in this study (Yang et al. 2002; Schmidt et al. 2007; Pandita et al. 2015).

Iron supply to the cells is critical for cell growth and development. Strict regulation of the endogenous iron metabolism in the developing fetus is justified by the need to provide the cells with the adequate amounts of iron to maintain vital biological processes such as oxygen transport, myelin and neurotransmitter synthesis, and DNA synthesis. Iron deficiency during the fetal or postnatal periods can alter brain structure, neurochemistry and cognitive functioning, and lead to long-term cognitive and motor impairment in later life, which cannot be reversed by iron supplementation. (Collard 2009; McArdle et al. 2011).

On the other hand, appropriate defense mechanisms are initiated to protect the fetus against the toxicity of oxidative stress mediated by iron excess (McArdle et al. 2011; Cao and O`Brien 2013). NGAL and Lf may limit iron-mediated cytotoxicity and also store iron released from damaged cells, by removing iron from the site of injury (Yang et al. 2002; Pandita et al. 2015).

It is unclear whether Lf and NGAL in fetal intestinal contents may be linked to the potent antibacterial activity of these proteins. The fetus and the intrauterine environment are no longer considered to be sterile with the initial microbiological exposure occurring only at birth. Microbiome may modify the environment of the developing fetus with possible short-and long-term impact on the individual`s heralth and disease. Meconium contains diversified microbiota derived from efflux of maternal commensal bacteria. It suggests that prenatal exposure of the fetal intestine could initiate intestinal colonization and might modulate immediate postnatal adaptation, including tolerance towards the colonizing bacteria. Maternal infection transmitted to baby in utero may be direct cause of the development of inflammation in the fetus, with the neutrophil involvement. Types of bacteria present in meconium may determine the development of the fetus’s immune system with important short-term and long-term health outcomes (Jiménez et al. 2008; Moles et al. 2013; Wilczyńska et al. 2017).

The iron endowed from maternal circulation is the main source of iron for infants until the age of 6 months (Black 2012; Cao and O`Brien 2013). The maintenance of iron homeostasis in the maternal-placental-fetal unit could include commonly recommended prophylactic iron supplementation in the mother with yet unclear consequences for the fetus. A question, of importance to clinical practice, arises whether meconium NGAL and Lf concentrations could be used as objective markers of optimal iron endowment of the fetus. According to the literature, increased iron supplementation may enhance NGAL expression at the fetal-maternal interface (Tadesse et al. 2012) It is also known that Lf may be responsible for hypoferremia due to local iron accumulation (Ward et al. 2003). Further studies are required to demonstrate the relationship between the concentration limits for meconium NGAL and Lf and the neonate’s optimal iron endowment followed by establishing the principles of safe iron supplementation during pregnancy.

Summing up, NGAL and Lf measured in meconium are candidate biomarkers for the assessment of homeostasis in the intrauterine environment of the unborn child. Further studies are needed to gain better understanding of physiological and non-physiological factors influencing the variability of NGAL and Lf concentrations in meconium associated with the fetal and maternal iron status. NGAL and Lf measured in meconium might be used to determine the optimal iron endowment of the fetus and to plan further therapeutic strategies when required to treat iron deficiency in the infant. Meconium naturally passed by a neonate can be easily and non-invasively obtained and is a potentially valuable source of specific biomarkers of intrauterine development.
